# Assessment of Urban-Rural Differences in Schizophrenia Mortality in the United States Using the CDC-WONDER Database: A Retrospective Study

**DOI:** 10.7759/cureus.89257

**Published:** 2025-08-02

**Authors:** Sylvester K Bote, Awab Adil Abdelaziz Mohamed Hamid, Aliona Chunarova

**Affiliations:** 1 Psychiatry, American University of the Caribbean, Cupecoy, MAF; 2 Psychiatry, Texila American University, Georgetown, GUY; 3 Psychiatry, Belarusian State Medical University, Minsk, BLR

**Keywords:** cdc-wonder, disparity, metropolitan, retrospective, rural, schizophrenia, urban

## Abstract

Introduction: Schizophrenia has been linked to a decrease in lifespan. Urban-rural disparity provides a snapshot of health risks within two separate populations and helps assess the population risks involved in schizophrenia mortality. This retrospective study aimed to assess disparities in mortality rates due to schizophrenia between urban and rural populations.

Methodology: This retrospective study analyzed schizophrenia-related mortality data (ICD-10 code F20) from 1999 to 2020, extracted from the CDC-WONDER Online Database on March 20, 2024. The data were stratified using the 2013 urbanization classification into urban and rural regions, and further categorized by age, sex, race, and census region. As a publicly available, de-identified dataset, it required no ethics approval. Statistical analysis was conducted using R (2023; R Foundation for Statistical Computing, Vienna, Austria), and visualizations were generated using GGPlot2.

Results: A total of 9,359 schizophrenia-related deaths were identified from 1999 to 2020 using the CDC-WONDER database. The absolute number of deaths was higher in urban areas (7,253) compared to rural areas (2,106). However, when adjusted for population, the crude mortality rate was significantly higher in rural areas across most demographic groups. This rural-urban disparity in the crude mortality rate was statistically significant for individuals aged 45 years and above, both sexes, and among the White and Black or African American populations (p < 0.001). Crude mortality rates in rural areas showed consistent elevation over time, despite fluctuations, and were notably higher in elderly age groups, particularly those aged 65-85+ years.

Conclusions: This retrospective study highlights significant differences in rural versus urban crude mortality rates in patients with schizophrenia and highlights the need to address disparities in healthcare for schizophrenia patients.

## Introduction

Schizophrenia is a severe psychiatric disorder and a heterogeneous behavioral and cognitive syndrome that is related to disturbances in brain development caused by hereditary or natural components [1]. In the United States alone, schizophrenia affects approximately 1.5% of the population, with an estimated mortality rate 2-3 times higher than that in the general population [2]. Trends in mortality associated with schizophrenia, particularly among younger age groups, have been noted, highlighting a persistent gap in life expectancy compared with the general population [3]. 

The urban-rural disparity in mortality rates is critical for public health policy and resource allocation, as differences in healthcare access, socioeconomic status, and environmental factors significantly impact health outcomes, including mortality rates [4]. Understanding the challenges faced by individuals with schizophrenia in urban versus rural settings is essential for developing tailored interventions to address their unique needs and to reduce disparities in mortality rates. Moreover, examining these disparities can provide insights into the broader social determinants of health affecting individuals with schizophrenia, informing strategies to improve overall health equity and reduce premature mortality. 

Despite the burden of schizophrenia and its disproportionate impact on mortality rates, there remains a prominent gap in research examining urban-rural disparities in mortality within this population [5]. While various factors contributing to premature mortality in individuals with schizophrenia have been explored, few studies have specifically investigated how these factors manifest differently in urban and rural contexts. Addressing this gap allows for a better understanding of the interplay between geographic location, healthcare access, and mortality outcomes in schizophrenia, potentially informing targeted interventions to improve health outcomes in diverse geographical settings. 

Aims and objectives

This research aimed to examine the differences in mortality rates between urban and rural regions among individuals with schizophrenia, utilizing data from the CDC-WONDER Online database. This study was designed to identify any significant associations between demographic variables and mortality rates. 

## Materials and methods

This retrospective original study was based on statistical extraction from the CDC-WONDER Online Database, a public health resource maintained by the Centers for Disease Control and Prevention (CDC) to provide access to de-identified health data. The materials were obtained on March 20, 2024 [6,7]. Data on schizophrenia-related mortality (ICD-10 code F20) were analyzed, focusing on records from 1999 to 2020.

The CDC-WONDER database sources its data from various national health surveys, vital statistics, and registries. Data collection and reporting processes adhere to rigorous standards to ensure accuracy, consistency, and protection of individual privacy. As a publicly available, de-identified dataset, its use in this study does not involve direct participation from human subjects, thereby exempting it from the requirement of ethics committee approval. Furthermore, the database complies with the Health Insurance Portability and Accountability Act (HIPAA) Privacy Rule, ensuring confidentiality and data security.

To gain a deeper understanding of the possible factors affecting schizophrenia mortality, the data were categorized by the complex nuances of urbanization patterns, using the 2013 urbanization data as a reference point [8,9]. The urban area was divided into four areas and there were two rural regions for analysis. Within the metropolitan area, there were four distinct regions: the Large Central Metro, Large Fringe Metro, Medium Metro, and Small Metro. Within the rural domain, two distinct regions were included: Micropolitan and non-core regions. Furthermore, to comprehend various aspects of the data, a detailed sorting into certain groups was performed by integrating several demographic parameters, such as age, gender, race, and census region. This detailed approach ensured an extensive analysis that closely explored the correlation between these variables, allowing for qualified insights into the epidemiological outlook related to schizophrenia mortality. 

Following thorough collection and analysis of the data, the material was exported to a Microsoft Excel spreadsheet, after which it was meticulously evaluated using a particular software. Statistical analysis was performed using R Core Team (2023; R Foundation for Statistical Computing, Vienna, Austria). The plot was created using GGPlot 2: Elegant Graphics for Data Analysis (Springer-Verlag, New York, 2016).

## Results

Aggregate data of 9,359 deaths from 1999 to 2020 were obtained for schizophrenia from the CDC WONDER database. 

Table 1 shows the absolute number of reported mortalities in urban and rural areas due to schizophrenia from 1999 to 2020 as per the 2013 Urbanization Classification. The total number of deaths due to schizophrenia in rural areas was 2,106, and that in urban areas was 7,253, from the year 1999-2020. 

**Table 1:** Absolute number of reported mortalities in urban and rural areas due to schizophrenia from 1999 to 2020 as per 2013 Urbanization Classification

Type	N (%)
Urban (Metropolitan area)	7,253
Large Central Metropolitan	2400 (33.1%)
Large Fringe Metropolitan	1825 (25.2%)
Medium Metropolitan	2010 (27.7%)
Small Metropolitan	1018 (14%)
Rural (non-metropolitan area)	2106
Micropolitan	1152 (54.7%)
Non-core	954 (45.3%)

**Figure 1:**
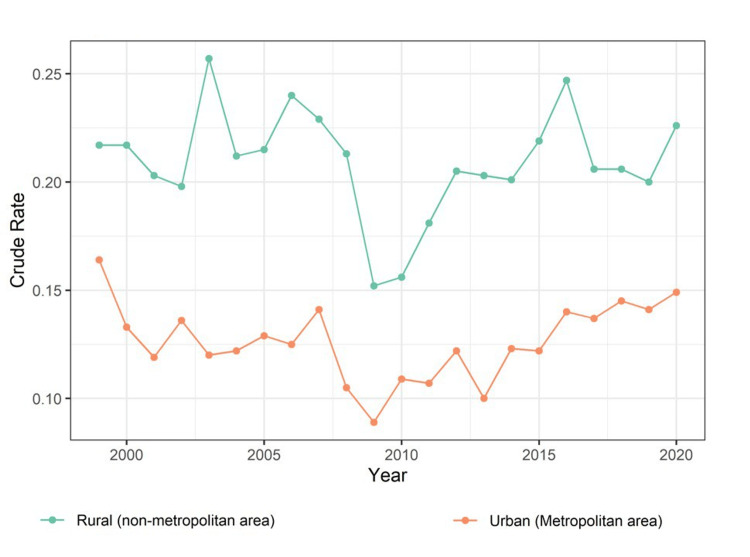
Line diagram showing trends in urban versus rural crude mortality rate due to schizophrenia calculated in the crude rate per 100,000 population

Figure 1 shows a line diagram of trends in urban versus rural mortality due to schizophrenia calculated in the crude rate per 100,000 population. The crude mortality rate in rural areas was consistently higher than that in urban areas. Mortality in rural areas showed some fluctuations throughout the years, but there were periods of increase and decrease. There appears to be a notable increase from 2002 to 2003, followed by some fluctuation and then a significant decrease from 2008 to 2009. The data also showed a notable increase from 2015 to 2016. The crude mortality rate in urban areas shows some fluctuations throughout the years with a significant decrease from 2007 to 2009. 

Table 2 shows the crude mortality rate due to schizophrenia in urban and rural areas based on age, sex, and race. The crude mortality rate due to schizophrenia is higher in rural areas than in urban areas across different age groups, sexes, and races. For instance, among individuals aged 55-64 years, the rural mortality rate (2.37 per 100,000) exceeded the urban rate (0.18 per 100,000). Both male and female rural populations experienced significantly higher mortality rates than their urban counterparts (p-values < 0.001). For example, the rural mortality rate for the female population (0.24 per 100,000) surpasses that for the male population (0.18 per 100,000). Similarly, the analysis of mortality rates across races reveals notable disparities, with rural areas consistently exhibiting higher crude mortality rates than urban areas. This is evidenced, for instance, by the Black or African American population in rural areas facing notably higher mortality rates (rural rate: 2.28 per 100,000) than in urban areas (urban rate: 0.141 per 100,000). 

**Table 2:** Crude mortality rate due to schizophrenia in urban and rural areas based on age, gender, and race *p-value < 0.005 was considered statistically significant

Variables	Urban		Mortality Rate (per 100,000)	Rural		Mortality Rate (per 100,000)	Binomial Test
	Mortality	Total population		Mortality	Total population		p-value
Age Groups							
< 1 year	NA	NA	NA	NA	NA	NA	NA
1-4 years	NA	NA	NA	NA	NA	NA	NA
5-14 years	NA	NA	NA	NA	NA	NA	NA
15-24 years	20	286359808	0.007	NA	NA	NA	NA
25-34 years	172	803054375	0.021	17	70755135	0.24	0.606
35-44 years	359	803916304	0.045	56	127369780	0.44	1
45-54 years	701	788609639	0.089	153	138965498	1.10	0.01*
55-64 years	1165	639136856	0.18	302	127286485	2.37	<0.001*
65-74 years	1684	417988177	0.40	493	92469123	5.33	<0.001*
75-84 years	1803	244183949	0.74	611	54320144	11.25	<0.001*
85+ years	1323	98324522	1.35	464	21189280	21.9	<0.001*
Gender							
Male	3392	2815055490	0.13	891	502292400	1.77	<0.001*
Female	3861	2924420159	0.13	1215	504579252	2.41	<0.001*
Race							
American Indian or Alaska Native	NA	NA	NA	NA	NA	NA	NA
Asian or Pacific Islander	120	345146399	0.035	NA	NA	NA	NA
Black or African American	1236	830766851	0.148778204	201	88267994	2.277156089	<0.001*
White	5866	4485984657	0.1307628191	1879	881053153	2.132674962	<0.001*

Associations between mortality rates and demographic variables were determined using binomial tests. Mortality rates were significantly associated with sex and race, while associations with age groups showed variations. The observed crude mortality rates varied across different age groups. Notably, individuals aged 65-74 years and 75-84 years’ experience higher mortality rates in both urban and rural areas. Conversely, mortality rates for younger age groups (e.g., 15-24 years, 25-34 years) exhibit less variation between urban and rural settings. As for sex, crude mortality rates were higher for women than for men in both urban and rural areas, with p-values indicating a significant association (p < 0.001). Additionally, Black or African American individuals face significantly higher crude mortality rates in both urban and rural contexts. The crude mortality rates for the white population also differ notably between urban and rural areas. 

## Discussion

A retrospective original research study was conducted to study the differences in the mortality of schizophrenia over 22 years. The data were drawn from CDC WONDER. 

Our study revealed that mortality from schizophrenia was higher in urban areas than in rural areas during the 22-year period from 1999 to 2020. Moreover, over the same 22-year period, schizophrenia mortality in urban areas was shown to be increasing, while mortality in rural areas decreased. According to age group analysis, mortality was significantly higher for age groups ranging from 45 to 85+ years. Gender disparity also leaned more towards a higher urban schizophrenia mortality for both men and women, with the African American population and the white population having significantly greater mortality from schizophrenia than other races. 

Dye et al. have shown that health disparities within differing populations are responsible for preventable mortality and morbidity [10]. As such, clarity through research on disparities helps identify key aspects of daily life that expose one population to increased death as opposed to another. These differences between urban and rural populations can be caused by multiple factors, including access to healthcare, lifestyle choices, poverty, and environmental factors. In the context of schizophrenia, which reduces the life span by 15-20 years, understanding disparities and individual risk factors may help increase the life span of these patients as well as those with greater schizophrenia mortality rates [3]. Additionally, these urban-rural mortality disparities indicate the need for specific policies to address the issues contributing to these disparities. An example of such policies is the provision of free access to health care to the less advantaged in urban areas that already have an increasing cost of living, which makes it hard to access health services. Increased mortality in the schizophrenic population has been linked to multiple comorbidities and unhealthy lifestyle choices [11]. Patients with schizophrenia have higher rates of metabolic syndromes and diabetes. They are also less likely to seek care for comorbid factors, ultimately leading to increased mortality [3,12]. Urban areas have been documented to have a higher number of schizophrenia cases, which are associated with greater mortality owing to an increased population with schizophrenia [13]. 

Luo et al. in their findings on a similar study on the association of urbanization and schizophrenia in China showed an increased risk of schizophrenia in urban areas; however, the mortality rate decreased with a higher degree of urbanization [13]. These changes were attributed to rural areas having limited access to emergency medical services compared with urban areas [14]. Compared to our data obtained from the United States of America, the Chinese study [13] had increased mortality in rural areas, which was also attributed to unequal development between different areas (urban vs. rural). These differences were less pronounced in the American land profile. Crump et al. in a Swedish cohort were also able to attribute increased mortality in schizophrenia to comorbidities including stroke, ischemic heart disease, cancer, and diabetes. The same study also found a strong relationship between schizophrenia and all-cause mortality in women [14]. Our study was able to dive deeper and found a significant increase in mortality in both genders in urban areas compared to the rural schizophrenic population. This increase is attributed to inadequate access to health care services in urban areas [15,16]. Hence, it is important for politicians and health-sector dignitaries to address these disparities to improve schizophrenic health outcomes in areas prone to higher mortality. Across genders, women attained increased mortality in both rural and urban populations, although only marginally. 

The CDC database has allowed for the collection of data that have made it easy to denote patterns in schizophrenia mortality. Although we have been able to acquire significant information, more work needs to be done to tease out information on the causes of these disparities. Multiple suggestions on the role of comorbidities have been made; however, prospective studies following these schizophrenic populations need to be conducted to gain a better insight into their lives and the intricate causes of mortality. Socioeconomic factors have been posited for mortality in rural areas in prior research; however, the economic landscape in America has changed with skyrocketing costs of living, particularly in urban areas. Hence, further work on the implications of these factors needs to be elucidated [4]. 

Limitations

This retrospective study used data obtained from the CDC WONDER website to test for disparities in mortality due to schizophrenia over a 22-year period from 1999 to 2020. Due to the coronavirus pandemic, data collection for the 2021-2023 period was severely hampered; hence, data for that period were not included in this study. Second, in the collection of data, we did not classify schizophrenia disorders based on their subcategories. Implications include a decreased sample size, although the disparity may have been mitigated by the length of the study. Lastly, causes of death specific to schizophrenia were not studied, as data were not included in the CDC WONDER. Healthcare and socioeconomic information were also not included, which might have painted a better picture of the possible causes of the disparities. 

## Conclusions

While urban areas had more deaths in absolute numbers, rural areas showed significantly higher crude mortality rates across most demographic groups. This disparity was particularly evident among individuals aged 45 and above, both sexes, and White and Black populations (p < 0.001).
